# Demographic profile and pattern of fatal injuries in Nairobi, Kenya, January–June 2014

**DOI:** 10.1186/s12889-016-3958-0

**Published:** 2017-01-06

**Authors:** Gladwell Koku Gathecha, Wilfred Mwai Githinji, Alfred Karagu Maina

**Affiliations:** Ministry of Health, Afya House, Nairobi, Kenya

**Keywords:** Injuries, Fatal, Mortality, Nairobi, Death certificates

## Abstract

**Background:**

Violence and Injuries are a significant global public health concern characterized by marked regional variation in incidence. Approximately five million people die from injuries each year, accounting 9% of all deaths worldwide. In Kenya, injuries are increasingly becoming a cause of hospital admissions and mortality where they account for 10% of all the deaths. The objective of this study was to determine the magnitude, demographic profile and pattern of fatal injuries in Nairobi.

**Methods:**

Retrospective review of death certificates from the Department of Civil Registration was done for deaths caused by injuries that occurred in Nairobi during the period, January to June 2014. Data was collected using a standardized form. Data entry, cleaning and analysis was done using Epi info version 7.0.

**Results:**

A total of 11,443 records were reviewed. From this data, deaths resulting from injuries were 1,208 accounting for 10.6% of all recorded deaths. Majority of the deaths resulting from injuries occurred in persons aged 25 to 44 years (48.1%). Males accounted for 85% of all the injuries. The leading cause of injury was assault by blunt force at 30.5%, followed by road traffic injuries at 25.9% and fire arm injuries at 15%. Pre-hospital deaths accounted for 51.4% of all the deaths. Nineteen percent of the deaths resulting from injuries had autopsies performed on them.

**Conclusion:**

Our study found that injuries are an important cause of fatality in Nairobi, accounting for one in ten deaths. There is need for multisectoral collaboration as some of the preventive measures that target the most prevalent injuries such as assault and road traffic injuries lie outside the health sector. There exists information gaps on the death certificates hence there is need to adequately capacity build both clinicians and death certifiers. There is also a need to revise the death certificates and to improve the pre-hospital care system for the injured persons.

**Electronic supplementary material:**

The online version of this article (doi:10.1186/s12889-016-3958-0) contains supplementary material, which is available to authorized users.

## Background

Violence and Injuries are a significant global public health concern characterized by marked regional variation in incidence [1]. It is estimated that violence and injuries account for 9% of all the global deaths, which is nearly 1.7 times the number of deaths resulting from HIV/AIDS, Tuberculosis and Malaria combined. Developing countries carry the largest burden of injuries and approximately 90% of the deaths due to injuries occur in low and middle income countries [2]. The high number of cases of injury fatalities among the less economically empowered communities can be attributed to unsafe environments, lack of effective prevention programs and poor access to quality health care.

Violence and Injuries result to considerable large number of hospital visits, hospital admissions and disabilities, and are a major cause of death. Injuries are responsible for 6% of all years lived with disability [2]. For every death related injury, there are a dozen more who get admitted, hundreds who visit the emergency department and a proportion of these will be left with a temporary or permanent disability [3]. Injuries also impose a huge financial burden among communities, which is incurred in form of direct costs of medical treatment or indirect costs as a result of loss of productivity. The true magnitude of the economic costs of violence and injuries remain largely unknown, but estimates for specific injuries such as road traffic injuries have been documented to cost nearly 3% of a Countries Gross National Product [4].

Prevention of violence and injuries is only possible when the magnitude and determinants of the two are well understood in a country. Unfortunately, injury data remains limited in Sub-Saharan Africa and Kenya is no exception [5]. There are numerous sources of data for violence and injuries; Civil Registration Data forms one of the key sources of information for violence and injury program planning [6].

Statistics from the National Transport and Safety Authority (NTSA) show that the fatality rate for road traffic crashes was 6.4/100,000 in 2015 [7]. A study carried out in a mortuary in Nairobi found that majority of the fatal injuries were caused by road traffic incidents (35%), followed by gunshot wounds (27%) and assault (20%) [8]. The Kenya Health and Demographic Survey (KDHS) 2014, revealed that 20 and 33% of women and men respectively had experienced a serious unintentional injury in the past 12 months. The common injuries among women as out lined in the KDHS were cuts (60%), falls (40%) and burns (20%). The common injuries among men were cuts (62%), falls (39%) and road traffic injuries (9%) [9].

The aim of this study was to determine the magnitude, demographic profile and patterns of fatal injuries in Nairobi County. We additionally sought to determine the proportion of fatalities attributable to injuries. There is limited data available on the magnitude and the leading causes of injury fatalities in Nairobi. This is the first study that details the magnitude and patterns of fatal injuries that is representative of Nairobi. Previous studies have described the fatal injury magnitude in selected areas such as the Nairobi City Mortuary hence the information cannot be generalized to the entire County [8]. This study explores the use of Civil Registration data by use of death certificates to detail the fatal injury burden, a method that has not previously been used. This information is particularly useful for policy formulation and implementation as it guides on prioritization of specific injuries and accompanying interventions. Our paper outlines the appropriateness of use of death certificates in a developing country like Kenya as a source of data for fatal injuries.

## Methods

This was a retrospective study done in Nairobi County which is the capital city of Kenya and has an estimated population of 3,138,369 in 2009 [10]. The growth rate of Nairobi has gradually increased, which is partially attributed to the increased number of informal settlements. The records review of death certificates was conducted at the Department of Civil Registration headquarters for deaths that had occurred in Nairobi County between January and June 2016. The Department of Civil Registration, under the Ministry of Interior Government, is responsible for the registration of all births and deaths in the Country. The system collects data from health facilities and local administrative units known as chiefs, who are responsible for notification of deaths that occur at home. Copies of death certificates are thereafter scanned and stored as both soft and hard copies at the headquarters.

The study population consisted of deceased persons whose death certificates were available and whose death was as a result of injury in Nairobi County between January and June 2014. Death certificates do not capture International Classification of Diseases (ICD) codes, but instead have the actual cause of death written and captured in a field titled “Cause of death”. The certificate also includes the antecedent causes of death, which look at morbid conditions that either led to the immediate cause or was an underlying cause. Other information includes demographics (age, sex, marital status and occupation) place and time of death. All ages were included in this study.

The records review was conducted by a trained research assistant. The soft copy records of the death certificates were reviewed and all the injury fatalities were recorded manually using the short version of the fatal injury surveillance data collection form, designed by World Health Organisation (WHO) and Monash University [11]. The data was then entered into an excel template prepared by WHO. Data cleaning and analysis was done using Epi info Version 7.0 software.

Descriptive analysis was performed to show distribution of the following key variables: Demographic variables (age and sex), mechanism of injury, place of injury, place of death and autopsy performed. Stratification of injury mechanism by age group and sex was done. The road traffic injuries were further analyzed to show the mode of transport and road user type. An injury case was defined as one whose primary cause of death was registered as an injury or violence in the death certificate. The mechanism of injury was how the injury was inflicted.

## Results

During the period January to June 2014, a total of 11,443 deaths occurred in Nairobi County. Deaths from injuries were 1,208 accounting for 10.6% of all the deaths. The demographic characteristics of the injury fatalities are detailed in Table 1. Males accounted for 85% of all the injuries. The mean age was 31.2 years. Majority of the injuries occurred in age group 25–44 years, accounting for nearly half of all the injuries (48.1%), followed by age group 20–24 years at 16.5%. The least affected age group was 5–9 years. Forty nine percent of the deaths occurred in hospital while the rest occurred at the injury site, on transit to hospital or were not specified. The records show that 19.2% of the fatalities had an autopsy performed.

**Table 1:** Demographic characteristics of all fatal injuries in Nairobi January–June 2014

Characteristic	*N* (%)
Sex	
Female	181 (15.0)
Male	1027 (85.0)
Age Group	
0–4	67 (5.6)
5–9	37 (3.0)
15–19	78 (6.5)
20–24	199 (16.5)
25–44	581 (48.1)
45–64	178 (14.7)
> 65	40 (3.3)
Place of death	
Hospital	588 (48.6)
Pre-hospital	620 (51.4)
Autopsy performed	
Yes	217 (19.2)
No	991 (81.8)
Total	1208

**Fig. 1:**
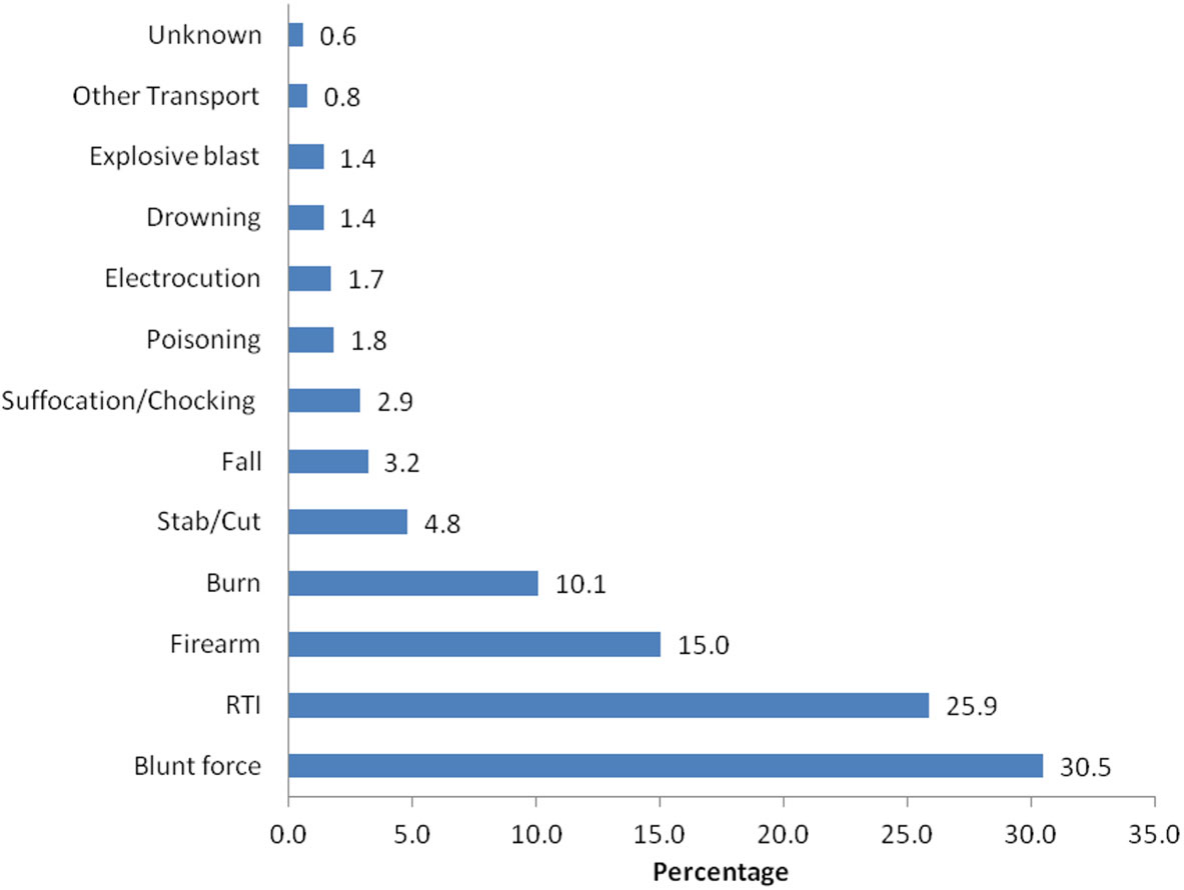
Mechanism of injury

Figure 1 illustrates that 30.5% of the fatal injuries were due to blunt force. Road traffic injuries recorded the second highest percentage at 25.9%, followed by firearm injuries (15.0%) and burns (10.1%). Explosive blasts and drowning both accounted for 1.4% of the deaths. The percentage of injuries that were unknown or unspecified was 0.6%.

Table 2 below shows the mechanism of injury by age group and sex. The highest cause of fatal injuries among the males was blunt force (32.4%), followed by road traffic incident (RTI) (24.6%), while among the females the highest cause of injury was RTI (33.1%) followed by burns (22.2%). RTI, blunt force, falls and burns were experienced in all the age groups. Among the age group 0–4 year in both sexes, burn injuries had the highest fatalities. No fatalities due to stab/cut, firearm and explosive blasts were recorded in children below 9 years. Females age 10–14 years had the least number of injuries. Majority of the males aged 15–19 died due to firearm injuries. Road traffic incidents were responsible for majority of the deaths among cases above 65 years. Twenty two percent of women died as result of burn injuries, the second leading cause of death in this group.

**Table 2:** Mechanism of injury by age group and sex

	0–4 *n*		5–9 *n*		10–14 *n*		15–19 *n*		20–24 *n*		25–44 *n*		45–64 *n*		65 + *n*		All Ages	
	M	F	M	F	M	F	M	F	M	F	M	F	M	F	M	F	Mn (%)	Fn (%)
Road Traffic Incident	5	2	1	7		4	6	1	38	9	140	21	49	10	14	6	253 (24.6)	60 (33.1)
Other Transport			1				1		2		3	1	1				8 (0.8)	1 (0.6)
Blunt force	6	3	6	5	5		20	2	59	4	175	12	52	7	10	2	333 (32.4)	35 (19.1)
Fall	2	3		5	2	1	1		4		15		8		3		35 (3.4)	9 (5.0)
Stab Cut							3		9		35	5	4	1	1		52 (5.1)	6 (3.3)
Drowning	3	2	2			2			1		6		1				13 (1.3)	4 (2.2)
Burn	15	14	3	0	4	2	4	5	9	1	32	12	8	1	2	5	77 (7.5)	40 (22.2)
Poisoning	3	2					2	1	1		9		4				19 (1.9)	3 (1.7)
Suffocation/Chocking	3	2	2	1	3		2	1	5	1	9	3	3				27 (2.6)	8 (4.4)
Electrocution	2		4		3		2	1			7	1					18 (2.6)	2 (1.1)
Firearm					2		25		50	1	82	4	15	2	1		175 (17.0)	7 (3.9)
Explosive blast								1	1	3	4	1	6	1			11 (1.0)	6 (3.3)
Unknown									1		4		1				6 (1.1)	0 (0.0)
Total	39	28	19	18	19	9	66	12	180	19	521	60	152	22	31	13	1027 (100)	181 (100)

A description of the 131 fatalities due to RTI according to road user type and mode of transport is given in Table 3 below. Six percent of the RTI fatalities occurred among pedestrians. Drivers and passengers accounted for 3.5 and 3.2% respectively. It was not possible to determine the type of road user in a large percentage of fatalities (87.5%). The mode of transport recording the highest proportion of fatalities was vehicle at 80.5%. Walking and the use of motorcycles accounted for 5.8 and 4.8% respectively. There were five fatalities that resulted from train incidents. It was not possible to determine the mode of transport in 7.3% of the records.

**Table 3:** Characteristics of Road Traffic Injuries Fatalities, January–June 2014

Characteristic	*N* (%)
Road User Type	
Pedestrian	18 (5.8)
Driver/Rider	11 (3.5)
Passenger	10 (3.2)
Unknown	274 (87.5)
Mode of transport	
Vehicle	252 (80.5)
Motorcycle	15 (4.8)
Train	5 (1.6)
Walking	18 (5.8)
Unspecified	23 (7.3)
Total	313 (100.)

## Discussion

Our study describes the magnitude and patterns of fatal injuries in Nairobi. The findings of this study are particularly useful because they give information on deaths that occur at both the community and hospital settings, hence quite representative. Furthermore, nearly a fifth of the deaths have autopsies performed on them and all death certificates are filled by a Medical Doctor as opposed to the rural areas where some certificates are filled by the Chiefs hence accuracy of the cause of death is relatively good.

Fatal injuries accounted for 10.6% of all the fatalities. This is similar to the estimated national percentage of injury mortality of 10% reported by WHO [12]. The burden of injuries in rural settings has been estimated to be lower than urban settings as demonstrated by Odhiambo et. al. who found that 4% of deaths were attributable to injuries [13]. While the global burden of injuries is on the decline [1], the burden of injuries in especially Sub-Saharan Africa remains high, possibly due to inadequate preventive measures, increased motorization and industrialization and weak health response [5]. Other East African Countries have recorded a higher percentage of injury fatalities in their capital cities. For instance, in Kampala Uganda, injuries account for 25% of the all the injuries [14] while in Kigali Rwanda, injuries account for 22% of all the deaths [15]. This difference may be attributed to the different sources of information that were used. Both studies utilized medical records but the Ugandan Study also included police records. Medical records have the disadvantage of not being population representative [16]. Our findings are comparable to a study carried out in Ghana that showed that 8.6% of the deaths were caused by injuries using mortuary records [17]. Undoubtedly, 10% is alarmingly high and while the Ministry of Health has prioritized reduction of Violence and Injuries as one of its policy objectives, a lot still needs to be done by both the Ministry and other sectors [18]. Coordination among all relevant sectors needs to be improved.

Our study found that majority of the injury fatalities occurred among the males at 85%. The sex ratio in Kenya for males: Females is 1:1.01. This huge sex disparity cannot be attributable to population profile but rather due to characteristics inherent in males [19]. Males have consistently been shown to be at a higher risk of injury and lifestyle and behavioral factors may be responsible [20,21]. The male proportion found is however larger than what has been reported in other studies; Rwanda 57% [15], Finland 75% [22] and Ethiopia 79% [23]. When compared across the age groups, males were still leading with the highest number of injury fatalities but this difference was less marked in those below 9 years. The mean age was 31.2 years. A study by Tsegaye et al. found a similar mean of 31 years among injury fatalities in Ethiopia [23]. The age group that was most affected was 25–44 years at 48.1%. This group however has the largest age group interval and it is therefore not surprising that it has the highest proportion. The second leading age group was 20–24 years at 16.5%. It should be noted that these two age groups are the most productive and the country stands to experience stagnation in economic growth and development if adequate measures are not put in place. The World Health Organisation further cites that injuries are the leading cause of death for young individuals between the age of 15–29 years particularly RTIs [2].

Slightly more than half (52%) of the deaths occurred outside the hospital. These findings are collaborated by other studies that have recorded a high percentage of pre hospital deaths [8,24]. While this may be an indication of the initial seriousness of the injury, it may also be due to other factors such as weak pre-hospital care systems [24–26]. The pre-hospital care systems in developing countries have been shown to be inefficient in infrastructure and human resources capacity as shown in a study by Wesson et al. [24]. In Kenya, devolution of health services including ambulance services took place in 2013 and sub-national entities sought to increase the number of ambulances in their jurisdiction. The biggest challenge however remains in standardization of both the ambulances and paramedic trainings. More studies are needed to evaluate the pre-hospital system in Nairobi such as the availability and effectiveness of the ambulance system and knowledge and skills of first responders.

The most prevalent mechanism of injury was blunt force at 31%. Blunt force injury was the leading cause of death among the males but was third leading cause among females. A study conducted in an informal settlement in Nairobi also found assault to be the leading cause of injury fatality [27]. Additionaly, another study in Western Kenya found that assault was the leading cause of injury fatality in men [13]. The homicide rate in Africa is the second highest in the world at 10.9 per 100,000 after the America’s region [28]. The high homicide rate in developing countries has been attributed to poor economic empowerment and lack of strong social cohesive networks [29]. Other African countries have reported that assault is the second leading cause of injury after road traffic injuries unlike the pattern in Kenya [14,15,23]. These finding points to a much deeper societal problem in our country due to youth unemployment and erosion of some of the more traditional African culture.

Three hundred and thirteen fatalities due to road traffic incidents (RTI) were recorded making it the second highest cause of injury fatality. All age groups were affected and RTIs were found to be the leading cause of death among females and among the age group 65 years and above. Despite Nairobi being an urban city that contains the highest number of vehicles in the country, RTI fatalities were not the leading cause of death. Our study found the recording of details pertaining to road traffic fatalities to be inefficient. It was not possible to determine the road user type and the mode of transport in 87 and 7% of the records respectively. This information is crucial for developing prevention programs [5]. With this challenge existing in the vital registration system, it has led the country to depend on police records, which under estimate the burden of road traffic fatalities in the country [30,31].

Police data has consistently shown over the years that majority of the road users affected by road traffic fatalities are pedestrians [7]. This discrepancy between our records and police data can be as a result of incompleteness and inaccuracies in capturing causes of death in vital registration systems. Vital registration systems have been acknowledged as the best method of recording road traffic injuries as they are able to capture deaths that occur at both the scene of crash and hospital [4]. Nevertheless, the death certification process is able to tell us that RTIs are an important cause of mortality that needs to be addressed.

Fifteen percent of the fatal injuries were caused by firearms. This affected persons older than 10 years only. Only seven females died as a result of firearm injuries compared to 175 males. Majority of fire arm injuries in Nairobi have been documented to be carried out by law enforcement officers [8,27] and it is likely a similar pattern occurred among these cases.

Burn injury fatalities accounted for 10% of the deaths. Zirapa et al., in a study conducted in an informal settlement in Nairobi found similar results of burn injury contributing to 9% of all the fatal injuries [27]. Burn injuries have been found to occur more in informal settlements [32]. The high number of informal settlements in Nairobi may be a huge contributor to this high proportion. Burns were the leading cause of injury fatalities for children below 5 year. The high burden of burn injuries among children suggest that continuous targeted education on close supervision to caregivers needs be implemented. According to the WHO, children below 5 years in Africa have five times the incident of burn fatality than any other region in the word and majority of the burns occur due to improper adult supervision [33]. Twenty two percent of the women died as a result of a burn injury making burn injuries the second leading cause of death in this group. It has been established that there is a higher burn incidence among females as they tend to do most of the domestic chores that involve the use of fire and hot objects [34]. Further studies are needed to explore the circumstances under which burn injuries occur.

Other injuries such as falls, chocking, poisoning and drowning recorded a low fatality incidence but are however of significant public health concern. The above mentioned injuries usually have high numbers in terms of morbidity as shown in hospital studies [35–37] and therefore preventive and control measures should be scaled up.

Our study was a first attempt at trying to determine the magnitude of injuries using Civil Registration data specifically in Nairobi and the Country at large. Sources of data for fatal injuries have been limited in the country. The study revealed several gaps in documentation and also identified areas of improvement. Information on the death certificates was in some instances incomplete. The use of ICD 10 codes would greatly improve reporting on cause of death and also improve on accuracy. A revision of the death certificates is additionally required so that RTI injuries are better captured in terms of road user and mode of transport. The Government is currently undertaking intensive trainings for death coders and certifiers to improve the quality of certification. A long term solution lies in the training of ICD-10 during undergraduate training as this is more sustainable.

## Limitations

The major limitation arising in this study is the lack of proper documentation of death certificates hence the missing out of key information. For instance, it was not possible to determine the intent of the injuries especially self harm since documentation on circumstance of death was in most cases missing. Injuries due to RTI were also not well documented to show a detailed disaggregation by type of road user and mode of transport. Although a death certificate is legally required for all deaths before burial, it is possible that some injury victims may have been buried without a death certificate due to various reasons. Some religions like Islam require that the dead be buried on the same day hence an expedited death certificate may not be possible depending on the time of death. Additionally, due to cultural practices surrounding the death, some communities do not request for death certificates. Lastly, the age group categorization used for analysis is not uniform and hence findings should be interpreted with caution as the age groups with a wider range may have resulted to recording of higher proportions than the age groups that had a smaller range.

## Conclusion

Our study found that injuries are an important cause of fatality in Nairobi accounting for one in ten deaths. There is need for multisectoral collaboration as some of the preventive measures that target the most prevalent injuries such as assault and RTI lie outside the health sector. We recommend that legislations, policies and guidelines should be strengthened to focus more on prevention of injuries because a significant proportion of deaths in Nairobi County are caused by preventable mechanisms. There exists gaps in information on the death certificates hence there is need to adequately capacity build both clinicians and death certifiers to improve the quality of data. There is also a need to revise the death certificates and to improve the pre-hospital care system for the injured persons.

## Additional file

Additional file 1: Fatal Injuries data_final. (XLS 2611 kb)

## Abbreviations

HIV/AIDS: Human immunodeficiency virus/acquired immune deficiency syndrome
ICD: International classification of diseases
RTI: Road traffic incident
WHO: World Health Organisation
